# Characteristics of imaging in hepatic inflammatory pseudotumors: a comparison between IgG4-related and IgG4-unrelated cases

**DOI:** 10.1186/s13244-024-01782-w

**Published:** 2024-08-09

**Authors:** Hua Lin, Ying Liu, Youyong Wei, Xiaohui Guan, Shuilian Yu, Yuping Man, Demao Deng

**Affiliations:** Department of Radiology, the People’s Hospital of Guangxi Zhuang Autonomous Region, Guangxi Academy of Medical Science, Nanning, 530021 Guangxi China

**Keywords:** IgG4, Inflammatory pseudotumor, Liver disease

## Abstract

**Objectives:**

The objective of this study was to examine the imaging features of hepatic inflammatory pseudotumors (IPTs) associated with IgG4-related and IgG4-unrelated conditions and to enhance the approach toward distinguishing between these two types of IPTs.

**Methods:**

A retrospective study was conducted, involving 20 patients diagnosed with hepatic IPTs. Imaging procedures were conducted within a timeframe of 4 weeks prior to hepatectomy or biopsy. The imaging features were then analyzed and compared using chi-squared analysis.

**Results:**

Seventeen (81.0%) IPTs were located in the hepatic subcapsular area; six (66.7%) IgG4-related IPTs were distributed around the hepatic hilum; and eleven (91.7%) IgG4-unrelated and three (33.3%) IgG4-related IPTs had unclear boundaries. All lesions exhibited similar characteristics in CT scans, T1-weighted imaging (T1WI), T2-weighted imaging (T2WI), and diffusion-weighted imaging (DWI), with the apparent diffusion coefficient (ADC) values slightly higher than the surrounding liver tissue. Delayed hypoenhancement, observed in five cases (55.6%), was exclusively present in IgG4-related IPTs. The remaining IPT lesions displayed progressive enhancement, septal and marginal enhancement, and persistent enhancement. Central enhancement was absent in three IgG4-related IPTs (33.3%) and ten IgG4-unrelated IPTs (83.3%). The duct-penetrating sign was identified in two IgG4-unrelated IPTs (16.7%) and seven IgG4-related IPTs (77.8%). Furthermore, seven patients with IgG4-related IPTs had additional lesions outside the liver.

**Conclusions:**

IgG4-related lesions are frequently found in the vicinity of the hepatic hilum; they display the duct-penetrating sign and affect other organs as well. Both groups exhibited progressive or persistent contrast enhancement in typical IPT lesions, but delayed hypoenhancement was only observed in the IgG4-related IPT group. IgG4-unrelated IPT lesions often exhibited indistinct boundaries lacking central enhancement.

**Critical relevance statement:**

Differences in imaging features differentiate IgG4-related and -unrelated inflammatory pseudotumors (IPT). IgG4-related lesions are frequently near the hepatic hilum, display duct-penetrating sign, and affect other organs. Only the IgG4-related group demonstrated delayed hypoenhancement. IgG4-unrelated IPT lesions often exhibited indistinct boundaries lacking central enhancement.

**Key Points:**

- Compared with IgG 4-unrelated IPTs, IgG4-related IPTs show delayed hypoenhancement and affect other organs.
- IgG4-unrelated IPTs have unclear boundaries and lack central enhancement.
- Improved IPT diagnostic capabilities can help minimize additional, potentially unnecessary, interventions.

**Graphical Abstract:**

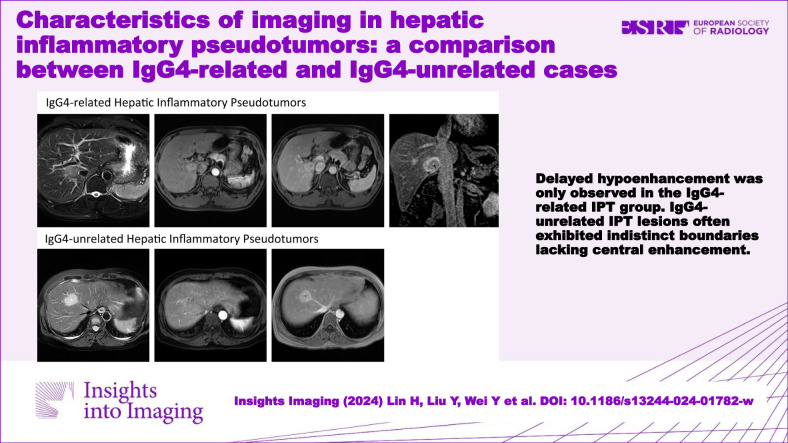

## Introduction

Inflammatory pseudotumors (IPTs) are non-cancerous growths characterized by the proliferation of fibrous connective tissue and infiltration of inflammatory cells. They are typically found in the lungs and rarely in other organs [1]. Although hepatic IPTs have been reported since 1953 [2], most published studies on these cases consist of individual reports [3, 4]. Diagnosis of hepatic IPTs is often delayed due to the absence of specific clinical and radiological signs, making it challenging to differentiate them from other focal liver lesions. Recently, hepatic IPTs have been categorized into two types: immunoglobulin (Ig) G4-related and -unrelated IPTs, based on their histological features [5]. A close relationship between IgG4-related immune responses and IPTs was found with many IgG4-positive plasma cells in IgG4 IPT biopsy samples and high serum IgG4 levels [6]. These two types differ in terms of pathological examinations, clinical presentations, morphology, locations, and treatment options [6]. Few reports exist on the imaging features of hepatic IPTs [7], and most of them were case reports. The lack of understanding of hepatic IPTs can resemble malignant tumors, often leading to a lengthy and expensive diagnostic process and sometimes unnecessary surgery. Therefore, we conducted a retrospective review of images from patients with confirmed hepatic IPTs to enhance understanding of the differential diagnosis and enable prompt management by improving diagnostic accuracy and providing guidance for treatment strategies.

## Materials and methods

### Study design and participants

We conducted a retrospective analysis of individuals who visited our hospital from October 2014 to October 2021. Our study focused on patients who met the following criteria: (1) had liver IPT confirmed through pathological examination, (2) underwent an abdominal CT or MRI scan within 4 weeks prior to the diagnosis of liver IPT, and (3) had no previous history of hepatectomy. We included patients who met these criteria and had complete medical records while excluding those with incomplete records (*n* = 2, images are incomplete or inaccessible on the PACS system) or unclear images (*n* = 1, the image has artifacts).

### Image capture and analysis

A CT scan was carried out using either a 64-slice Siemens spiral CT scanner or a 256-slice Philips spiral CT scanner. Parameters: 120 kV, 300 mAs. The enhanced CT scan was conducted using a contrast agent called iopromide. The contrast agent had a concentration of 300 mg/mL, a total volume of 75 mL, and a flow rate of 3.5 mL/s. Images were acquired at 25, 60, and 180 s after the injection to capture the arterial-phase, portal-phase, and delayed-phase scans, respectively. The MRI was conducted using either a Siemens Avanto 1.5T or a Siemens Skyra 3.0T. The specific settings employed were as follows:

1. An axial fat-saturated fast spin echo T2WI sequence (TR/TE = 3000/69 ms).
2. An axial gradient echo sequence was employed, including positive- and negative-phase T1WI (TR/TE = 4.11 ms/2.54, 1.31 ms).
3. A coronal T2WI configuration was used with a coaxial-position T2WI.
4. A DWI axial-position (TR/TE = 1800/47 ms), b-value (50, 800).
5. An enhanced scan: Gd-DTPA contrast medium (0.1 mmol/kg, 2 mL/s) was administered. The portal, venous, and delayed phases were recorded at 15, 90, and 180 s after the arterial phase.

Two senior radiologists independently examined all the images. They provided details about the IPT lesions, such as their size, shape (round, lobulated, or irregular), boundary (clear or unclear), number, location (left and right lobes), and distribution in the hilum and subcapsular regions. Compared with the surrounding liver tissue, the densities of the non-contrast-enhanced CT images were categorized as slight hyperdensity, isodensity, or slight hypodensity; the signals observed on MRI T2WIs were classified as hyperintense, isointense, or hypointense. The signal or density after the enhanced scans was described as diffuse homogeneous enhancement, diffuse heterogeneous enhancement, circumferential enhancement, or no enhancement. The dynamic enhancement patterns were characterized as progressive (gradual increase in enhancement over time), persistent (consistent enhancement in all three phases), or washout (initial arterial enhancement followed by hypodensity in the portal or delayed phase). Any other coexisting abnormalities were also documented, such as central non-enhancement, delayed-phase capsule-like enhancement, septal enhancement, bile duct dilatation, and abnormal perfusion in the surrounding liver tissue. Delayed-phase capsule-like enhancement refers to a specific type of ring-shaped enhancement along the lesion’s edge during the portal or delayed phase. The apparent diffusion coefficient (ADC, ROI size: 20 mm^2^) values of the lesion and the adjacent normal liver tissue were measured in the same slice while avoiding large vessels, bile ducts, and necrotic tissues for the selection of the region of interest.

### Pathology analysis

The hepatic IPTs were classified into two types, namely IgG4-unrelated (fibrohistiocytic) and IgG4-related (lymphoplasmacytic), based on their histologic features [6]. IgG4-unrelated IPTs displayed characteristics such as xanthogranulomatous inflammation, multinucleated giant cells, and infiltration of neutrophils. On the other hand, IgG4-related IPTs were identified by the presence of diffuse lymphoplasmacytic infiltration, which included an increase in the number of IgG4-positive plasma cells, a ratio of IgG4-positive/IgG-positive plasma cells greater than 40%, and/or a higher count of IgG4-positive plasma cells exceeding 50 per high-power field (HPF) in surgical specimens or more than 10/HPF in biopsy specimens. Additionally, IgG4-related IPTs were characterized by fibrosis, typically displaying a storiform pattern, and obliterative phlebitis [8].

### Statistical analysis

The chi-squared analysis was used to compare categorical variables. The two consecutive sets of variables were compared using a paired *t*-test. All statistical analyses were conducted using SPSS 22.0 software (IBM Corporation located in Armonk, NY, USA).

## Results

### Study participants and characteristic comparisons

**Fig. 1:**
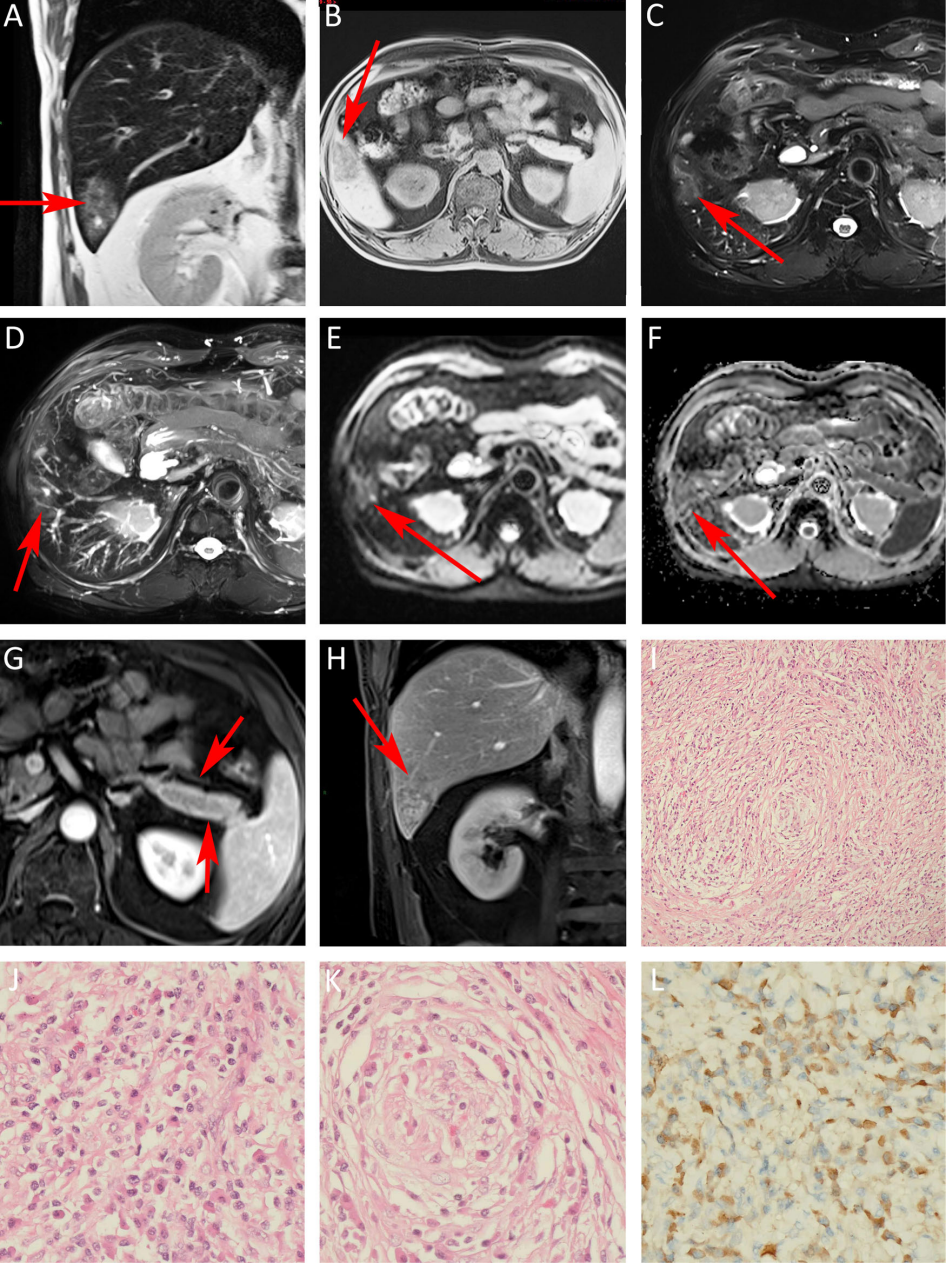
A 63-year-old man has an IgG4-related IPT. **A**, **C**, **D**, **H** FIESTA and T2WI MIP reveal duct-penetrating sign. **B** A lesion with slightly hypo-intense signals in T1WI. **E**, **F** DWI and ADC demonstrate slightly brighter signals. **G** The edge of the pancreas was smooth and blunt, and the internal fine structure disappeared, showing a salami appearance. The thin line halo sign can be seen in front of the tail of the pancreas. **I**, **J** H&E laminated hyperplasia of fibrous tissue and infiltration of plasma cells. **K** Obliterative phlebitis. **L** Immunohistochemistry IgG4-positive plasma cells

This study included a total of 20 patients with hepatic IPTs. The epidemiological and clinical features of this study are shown in Table 1. All diagnoses in this study were confirmed through surgery or biopsy evaluations. The study is divided into IgG4-related and IgG4-unrelated hepatic IPT groups, and their distribution is shown in Table 2. Among them, eight patients had IgG4-related hepatic IPTs, consisting of seven men and one woman with an average age of 56 years (age range: 43–77 years). These patients had a total of nine lesions. Hepatitis B was not detected in these patients with abnormal liver function. Seven patients also had involvement of other organs (such as bile duct, gallbladder, pancreas, kidney) (Fig. 1G). Furthermore, there were twelve patients with IgG4-unrelated hepatic IPTs, comprising nine men and three women with an average age of 50 years (age range: 13–73 years). They had a total of twelve lesions. Only three patients in each group underwent DWI scans.

**Table 1:** Comparisons of clinical characteristics and laboratory results between patients with IgG4-related and -unrelated hepatic IPTs

Variables	IgG4-related IPT group	IgG4-unrelated IPT group	*p* value
Gender, M/F	7/1	9/3	> 0.05
Age, year, mean	56	50	> 0.05
Clinical presentations	Abdominal pain (2), jaundice (3), or incident finding (3)	Abdominal pain (8), and/or fever (2), incident finding (3)	
Abnormal hepatic function	4	1 (except cirrhosis and hepatitis B)	< 0.05
Mildly elevated tumor markers	4	1	< 0.05
Concurrent lesions in other organs	7	0	< 0.05

**Table 2:** Comparisons of imaging characteristics between patients with IgG4-related and -unrelated hepatic IPTs

Variable	IgG4-related IPT group	IgG4-unrelated IPT group	*p* value
Unclear boundary	3 (33.3%)	11 (91.6%)	< 0.05
Subcapsular distribution	7 (77.7%)	10 (83.3%)	> 0.05
Surrounding hepatic hilum	6 (66.6%)	0	< 0.05
Right or left hepatic lobe	Right (6)/left (3)	Right (10)/left (2)	> 0.05
Progressive enhancement	3 (33.3%)	6 (50.0%)	> 0.05
Persistent enhancement	3 (33.3%)	4 (33.3%)	> 0.05
Delayed hypoenhancement	5 (55.5%)	0	< 0.05
Rim enhancement in the delayed phase	1 (11.1%)	2 (16.6%)	> 0.05
Enhancement of liver parenchyma surrounding the lesion	1 (11.1%)	4 (33.3%)	> 0.05
No central enhancement	3 (33.3%)	10 (83.3%)	< 0.05
Duct-penetrating sign	7 (77.7%)	2 (16.7%)	< 0.05
Non-contrast-enhanced CT density	8 slightly lower	9 slightly lower	> 0.05
T1WI	9 slightly lower	8 slightly lower	> 0.05
T2WI	8 slightly higher	8 slightly higher	> 0.05
DWI	ADC values higher than the liver tissue in three lesions (100%)	ADC values higher than the liver tissue in three lesions (100%)	> 0.05

Among the IgG4-related group, seven received both CT and MRI scans, one patient only received CT scan. Among the IgG4-unrelated group, five received both CT and MRI scans, four had only CT scans, and three had only MRI scans. Only three patients in each group underwent DWI scans

Table 1 demonstrates that there were no significant differences in age and gender distribution between the two groups. However, statistically significant differences were observed in terms of liver function, tumor markers, and concurrent involvement of other organs.

### Imaging characteristics

**Fig. 2:**
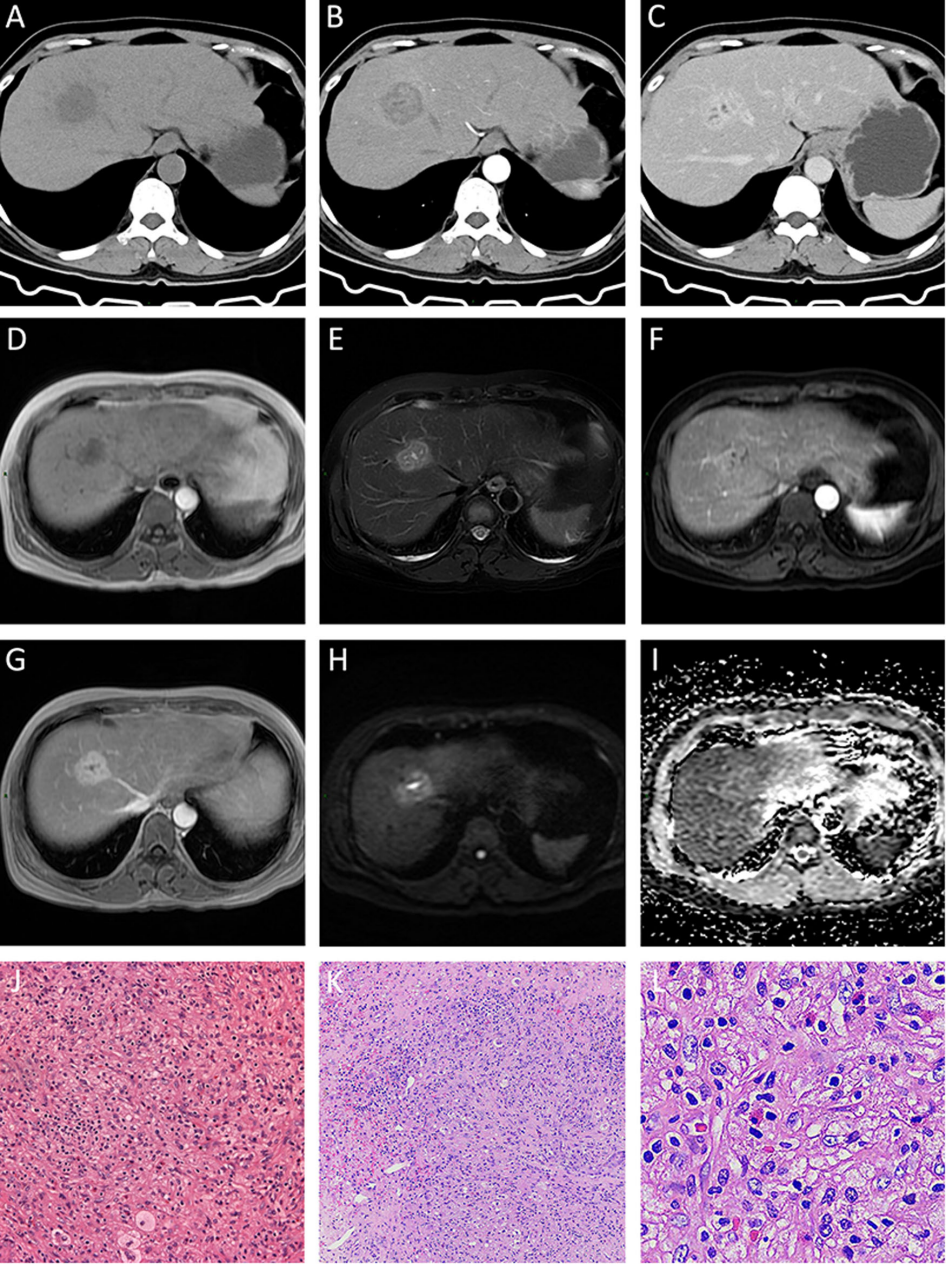
A 46-year-old woman has an IgG4-unrelated IPT. **A** CT image shows a slightly hypodense lesion with unclear boundaries. **D**, **E** A lesion with slightly hypo-intense signals in T1WI and slightly hyper-intense signals in T2WI, with unclear boundaries. The lesion exhibits progressive enhancement from the arterial to delayed phases (**B**, **C**, **F**, **G**) with central non-enhancement. Both DWI and ADC (**H**, **I**) show slightly brighter signals. **J**, **K**, **L** Fibroblast proliferation in a background of macrophages, along with notable lymphocyte infiltration and scattered eosinophil infiltration

**Fig. 3:**
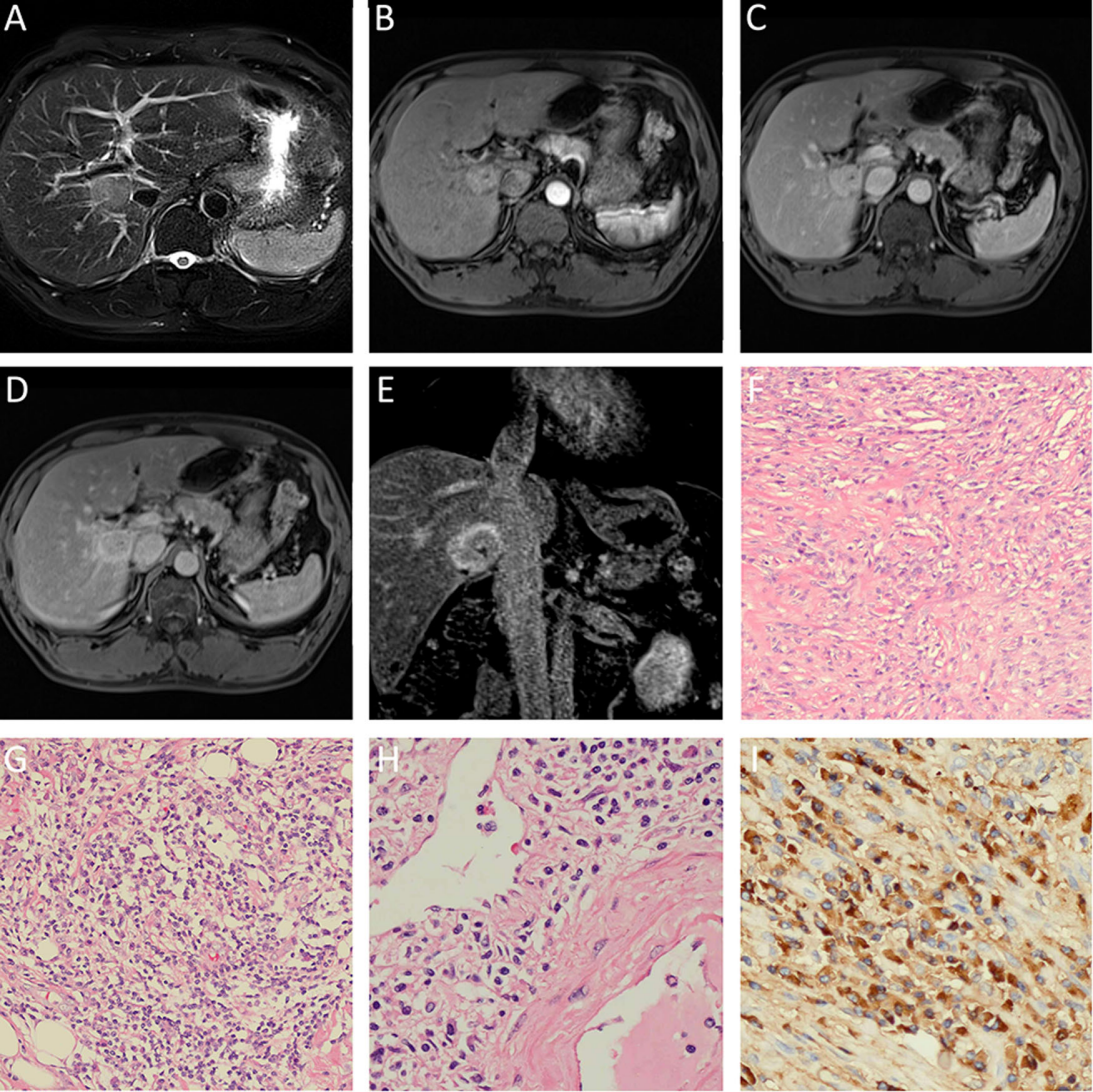
A 43-year-old man with IgG4-related IPT. **A** Duct-penetrating sign can be observed. **B**, **C**, **D** and **E** demonstrate enhancement and washout in the venous and delayed phases. Rim enhancement and duct-penetrating sign can be found. **F** H&E staining layered fibrous tissue hyperplasia. **G** Massive infiltration of lymphocytes and plasma cells. **H** H&E, phlebitis through. **I** Immunohistochemistry staining, substantial infiltration of IgG4-positive plasma cells

The lesions varied in size, with the maximum diameters ranging from 1.2 to 7.2 cm. On average, the lesions had a diameter of 3.3 cm. These lesions were either round or oval in shape and did not show any noticeable lobulations. Table 2 presents the details of the lesion distribution, boundary, density, and other image characteristics. The majority of IPTs (81.0%) were found in the hepatic subcapsular area, while 66.7% of IgG4-related IPTs were distributed around the hepatic hilum. Unclear boundaries were observed in 91.7% of IgG4-unrelated IPTs and 33.3% of IgG4-related IPTs. All lesions exhibited decreased attenuations on CT scans, with low-intense signals on T1WI and slightly high-intense signals on T2WI (except for one IgG4-related IPT, which had slightly low-intense signals) (Figs. 2E, 1C, and 3A). Dynamic contrast-enhanced scans revealed that 33.3% of IgG4-related IPTs and 83.3% of IgG4-unrelated IPTs did not exhibit central enhancement (Fig. 1G). IgG4-unrelated IPTs showed different enhancement patterns: 50.0% had progressive homogeneous enhancement (Fig. 2F, G), 33% had septal and marginal enhancement, and 17.6% had persistent enhancement. IgG4-related IPTs displayed progressive enhancement in 33.3% of cases (Fig. 1G, H), persistent enhancement in 11.1% of cases, and delayed hypoenhancement in 55.6% of cases (Fig. 3B–E). Rim enhancement in the delayed phase was observed in 16.7% of IgG4-unrelated IPTs and 11.1% of IgG4-related IPTs (Fig. 3E). Surrounding liver parenchyma enhancements were present in 33.3% of IgG4-unrelated IPTs and 11.1% of IgG4-related IPTs. The duct-penetrating sign was seen in 16.7% of IgG4-unrelated IPTs and 77.8% of IgG4-related IPTs (Figs. 1D, H, 3A, E). DWI showed slightly high-intense signals for all lesions, and their ADC values (100%) were slightly higher than those of the surrounding liver tissue, with a mean ADC value of approximately 1.359 ± 0.32 × 10^−3^ mm^2^/s (Figs. 1F and 2I).

## Discussion

In our study, the occurrence of both types of hepatic IPTs was more prevalent in middle-aged and elderly men, which contrasts with the pattern observed in other autoimmune diseases that primarily affect women [9, 10]. Hepatic IPT has no specific epidemiological or clinical characteristics, and accurate diagnosis remains a medical challenge. Therefore, imaging, especially MRI, is necessary and may play a key role in diagnosis.

The MR Imaging of IPT has certain characteristics in our study. Both groups exhibited progressive or persistent contrast enhancement in typical IPT lesions, which can be explained by a rich infiltration of inflammatory cells in the center and a concentrated fibrous tissue around it [11]. However, delayed hypoenhancement (contrast medium washout) was only observed in the IgG4-related IPT group (*n* = 5, 55.6%), which is consistent with some literature reports [12]. Kong et al proposed that this delayed hypoenhancement may be attributed to concurrent obliterative phlebitis, fewer venous vessels within the lesion, and inadequate blood supply to the portal vein [13]. Delayed rim enhancement was less frequent (*n* = 3, 14.3%), which aligns with the findings reported in the existing literature. Rim enhancement could be a result of the presence of abundant fibrous tissues surrounding the lesion, as no true capsule was identified in the gross specimens or microscopic examination [12]. The absence of central enhancement was more commonly observed in the IgG4-unrelated IPT group (*n* = 10, 83.3%), the same as the study from Calistri et al [7]. This could be attributed to associated focal coagulative necrosis, abscess formation, and dilation of intrahepatic bile ducts with a dense central cellular component [11, 13]. The duct-penetrating sign—“blood vessel or bile duct penetration”, a distinctive characteristic of autoimmune pancreatitis [14], was seen in some literature on hepatic IPT [15] and in our study. Inflammation tends to be intense and primarily affects the submucosa area of the bile duct while sparing the bile duct epithelium and inner wall [6, 9, 16]. In the IgG4-unrelated IPT group, the structure of the bile duct was often compromised. In this study, the duct-penetrating sign was more prevalent in the IgG4-related IPT group (*n* = 7, 77.8%) compared to the IgG4-unrelated IPT group (*n* = 2, 16.7%), with a statistically significant difference.

On DWI, appearance at high b-values (no lower than the value of surrounding liver tissue) is reported in 41% of inflammatory pseudotumors of the liver (IPTLs) in the literature data (23/56 lesions) vs. 100% of our series (6/6 lesions). Our result is identical with Calistri et al [7].

Additionally, we found that six patients (75%) with IgG4-related IPTs also exhibited systemic involvement, such as parotid gland, autoimmune pancreatitis, retroperitoneal fibrosis, renal lesions, and intra- and extrahepatic biliary stenosis. IgG4-related illnesses encompass a group of chronic autoimmune diseases that can affect multiple organs and systems in the body. The pancreas and hepatobiliary system are the most frequently involved organs, either simultaneously or sequentially [17].

The majority of the IPT lesions in both groups were found in the subcapsular region and the right lobe of the liver. Most of the IgG4-related IPT lesions (*n* = 6, 66.7%) were distributed around the hepatic hilum. Existing research has also indicated that IgG4-unrelated IPTs are commonly situated in the liver tissue, while IgG4-related IPTs are typically found around the hepatic hilum and along the bile ducts near the hilum [6].

In our study, most of the IgG4-unrelated IPTs (*n* = 11, 91.7%) had indistinct boundaries, which could have caused subjective symptoms leading to early-stage diagnosis in most patients. At an early stage, the IPT lesions displayed infiltration of inflammatory cells and congestion and swelling of the surrounding tissues. As the disease progressed, accompanied by the proliferation of fibrous tissue around the periphery. During the recovery stage, most lesions exhibited proliferation and the formation of granulomas [18, 19]. In contrast, the majority of IgG4-related IPTs were diagnosed in patients without symptoms or with jaundice when the lesions were already in the recovery stage, showing the proliferation of fibrous tissue and clear boundaries.

Patients with IgG4-unrelated hepatic IPTs commonly experienced subjective complaints of epigastric discomfort and fever (75.0%), whereas those with IgG4-related IPTs often exhibited asymptomatic jaundice (75%). These findings align with a previous report [6]. In our study, significant differences were observed in abnormal liver function test results and positive CA199 findings. Patients typically present with jaundice and abnormal liver function test results due to biliary duct involvement, which commonly leads to stenosis in the lower segment of the common bile duct. A slight elevation in CA199 levels could also aid in distinguishing IgG4-related IPTs from other conditions [20].

### Differential diagnosis

Differentiating liver IPT from common liver lesions is important. In hepatocellular carcinoma (HCC), there is a non-peripheral washout, an enhanced capsule, the ADC value is lower, and it typically lacks a central scar on imaging [21]. It is often associated with hepatitis B or cirrhosis and shows elevated AFP levels. Some IgG4-related IPTs may have washout, which is difficult to distinguish from HCC. ADC values may help in forming the differential diagnosis. However, the number of patients undergoing DWI examination is small, and more cases need to be studied.

Cholangiocarcinoma [22] often shows a significant increase in CA199 levels and “slow in and slow out” mode during an enhanced scan. ADC values are lower, and the presence of dilated bile duct and bile duct amputation sign are characteristic features. Other imaging features of cholangiocarcinoma are membrane contracture and progressive enhancement of HBP.

Hepatic epithelioid hemangioendothelioma frequently shows a target appearance on T1WI/T2WI/DWI, a ring or target sign on the dynamic study similar to IPTL. However, hepatic epithelioid hemangioendothelioma more often shows a peripheral distribution of nodules (multiple), with coalescence and capsular retraction [23].

Metastatic liver cancer typically presents with multiple lesions, a history of a primary tumor, and elevated tumor markers. It exhibits a “target sign” on enhancement due to a necrotic center and surrounding viable tumor with central T1/T2 hypo/hyperintensity and peripheral T1/T2 hyper/hypointensity. Moreover, all metastatic lesions, demonstrate peripheral arterial ring enhancement because of peritumoral desmoplastic reaction [24].

In liver abscesses [24, 25], central fluid necrosis shows limited diffusion on DWI, lower ADC values compared to normal liver, and ring enhancement. The central fluid necrosis is not enhanced in the diffusion-limited area, and the cyst wall shows significant enhancement with no restricted diffusion.

Hepatic hemangioma [26] usually has no clinical symptoms and appears as a “bulb sign” on T2WI. The contrast agent gradually fills the lesion from the periphery to the center in a “fast-in and slow-out” mode during an enhanced scan.

Surgery is generally not necessary for patients with liver IPT. Non-IgG4-related IPT is usually treated with anti-inflammatory therapy, while IgG4-related IPT is treated with steam treatment. Analyzing the clinical and imaging features of the lesions is crucial for timely diagnosis, avoiding misdiagnosis, mistreatment, and unnecessary surgical procedures.

### Limitations

The retrospective nature of our study and the small number of participants pose limitations. Only a limited number of patients underwent the DWI examination, and the ADC values obtained differed significantly from those reported in previous literature [16]. To delve deeper into the imaging traits of IPT lesions, it is necessary to conduct multi-center studies with larger sample sizes.

## Conclusions

In conclusion, images of hepatic inflammatory pseudotumors (IPTs) exhibit distinct characteristics. These lesions are typically located subcapsularly within the liver. On T1-weighted imaging (T1WI), they present as low signal areas, while on T2-weighted imaging (T2WI), they exhibit slightly high signal intensity. Diffusion-weighted imaging (DWI) reveals high signal intensity, and the apparent diffusion coefficient (ADC) values are slightly higher than those of the surrounding liver tissue. Multi-phase enhancement scans predominantly show progressive or continuous enhancement patterns.

Comparatively, IgG4-related hepatic IPTs possess specific features distinct from IgG4-unrelated hepatic IPTs. They are often distributed around the hepatic hilum with relatively clear boundaries. In the IgG4-related group, delayed enhancement washout is commonly observed. These lesions frequently exhibit a duct-penetrating sign, with symmetrical, uniform, and slightly thickened and enhanced bile duct walls. Furthermore, IgG4-related hepatic IPTs are frequently associated with other systemic manifestations, such as autoimmune pancreatitis, retroperitoneal fibrosis, renal lesions, and thickening of the bile duct and gallbladder walls. Elevated serum IgG4 levels may also serve as a valuable diagnostic indicator.

## Data Availability

The datasets used and/or analyzed during the current study are available from the corresponding author upon reasonable request.

## Abbreviations

Gd-DTPA: Gadolinium-diethylenetriaminepentaacetic acid
HPF: High power field
IPT: IgG4-unrelated hepatic inflammatory pseudotumor
T1WI: T1-weighted imaging
T2WI: T2-weighted imaging
TE: Echo time
TR: Repetition time
